# 589 Video-Enhanced Telepresence for Burn Care may Improve Patient and Staff Satisfaction

**DOI:** 10.1093/jbcr/irac012.217

**Published:** 2022-03-23

**Authors:** Alfredo C Cordova, Chelsea Kane, Samuel M Miller, Margaret Heller, Nicole O Bernal

**Affiliations:** The Ohio State University Wexner Medical Center, Dublin, Ohio; The Ohio State University Wexner Medical Center, Columbus, Ohio; Yale University School of Medicine, New Haven, Connecticut; The Ohio State University Wexner Medical Center, Columbus, Ohio; The Ohio State University Wexner Medical Center, Columbus, Ohio

## Abstract

**Introduction:**

Telemedicine and telepresence technology successfully contributes to the care of patients in different medical disciplines. Burn care is a highly specialized field that requires a multidisciplinary team and frequent visual evaluation of a patient’s injuries. Wound care can be extensive and demanding. Physician availability at the “Wound Care Room” is often limited by other responsibilities in the operating room, emergency department, surgical clinic, or academic conferences. We hypothesized that the incorporation of a telemedicine platform would provide greater access to surgical providers (SP- burn surgeons, general and plastic surgery residents) and allow for more efficient evaluation and prompt decision making. We also predicted that it would improve communication among SP, nursing staff (NS- nurses and nurse aides), and burn patients (BP) in real time without compromising BP privacy and comfort.

**Methods:**

A dual-way video and voice telemedicine platform was incorporated into burn care at a Level-1 Trauma Center and ABA-verified Burn Center. The video module was positioned so that SP were able to remotely assess the progression of burn injuries during wound care. Patients included were hospitalized and undergoing wound care by NS. Adult BP were included regardless of age, burn thickness, and burn surface area. BP, SP, and NS were asked the following questions after wound care had been provided:

1. Did you feel comfortable using this technology?

2. Was the patient’s sense of privacy compromised?

3. Did use of the video module enhance the provision of care?

4. Did use of the video module improve team communication?

5. Overall, were you satisfied with the use of the platform?

**Results:**

BP with ages ranging from 18-74 years old and with injuries involving 8-44% TBSA were included. Interviews from 38 patient encounters were conducted, and included input from 4 SP, 6 NS, and 24 BP.

The BP, SP, and NS surveyed all reported comfort using this technology. There were no reports of concern for patient’s privacy. SP felt they could make a final management plan in 74% of the cases, with difficulty arising in 26% of cases due to image resolution. BP reported that use of the video modules contributed positively to their care in 87% of cases, with issues related to communication and lack of understanding arising in the other 13%.

**Conclusions:**

Telemedicine was well accepted by all the BP, SP, and NS. The perception from NS and SP was that it enhanced prompt communication contributing to better patient care. Final management decisions were achieved in most cases, with picture resolution being identified as an area for improvement. With improved picture quality, this technology can likely be used as a reliable decision-making tool to improve care.

